# Gender differences in prevalence and clinical correlates of anxiety in first-episode and drug-naïve patients with major depressive disorder comorbid with metabolic syndrome

**DOI:** 10.1186/s12888-024-05574-w

**Published:** 2024-02-22

**Authors:** Wenqi Gao, Zhifang Deng, Xiaonan Cai, Dan Zhang, Han Xiao, Xiangyang Zhang

**Affiliations:** 1 Institute of Maternal and Child Health, Tongji Medical College, Wuhan Children’s Hospital (Wuhan Maternal and Child Healthcare Hospital, Huazhong University and Technology, 430015 Wuhan, Hubei China; 2 Department of Pharmacy, Tongji Medical College, The Central Hospital of Wuhan, Huazhong University of Science and Technology, 430030 Wuhan, China; 3 Woman healthcare department for community, Tongji Medical College, Wuhan Children’s Hospital (Wuhan Maternal and Child Healthcare Hospital, Huazhong University and Technology, 430015 Wuhan, Hubei China; 4 CAS Key Laboratory of Mental Health, Institute of Psychology, Beijing, China; 5 Department of Psychology, University of Chinese Academy of Sciences, Beijing, China

**Keywords:** Metabolic syndrome, Anxiety, First-episode and drug-naïve major depressive disorder, Chinese han, Gender differences

## Abstract

**Background:**

Although gender differences in major depressive disorder (MDD) have been widely reported, there has not been much focus on gender differences in comorbidity. In patients with MDD and comorbid metabolic syndrome (Mets), the goal of this study was to investigate potential gender differences in the prevalence and clinical correlates of concomitant anxiety.

**Methods:**

Seven hundred and ninety-four first-episode and drug-naïve patients (FEDN) patients with MDD and comorbid Mets were recruited. For each patient, sociodemographic data, thyroid function indicators, and Mets parameters were acquired. Each participant completed the 14-item Hamilton Assessment Scale for Anxiety (HAMA) and the 17-item Hamilton Assessment Scale for Depression (HAMD).

**Results:**

There were no gender differences in the prevalence of anxiety in patients with MDD and comorbid Mets. Female patients with MDD had a shorter duration of illness. Correlation analysis showed that HAMD score, TSH, TgAb, and TPOAb were associated with anxiety prevalence in female patients, whereas anxiety onset in male patients was only associated with TSH, TgAb, and TPOAb levels. In addition, multiple logistic regression analysis showed that TSH and TgAb predicted anxiety in male patients, whereas HAMD score and age of onset significantly predicted anxiety in female patients.

**Limitations:**

Cross-sectional design and no control for anxiety-related factors.

**Conclusions:**

Our study showed no gender differences in the prevalence of anxiety in patients with MDD and comorbid Mets. HAMD score was associated with anxiety in female patients, whereas TSH, TgAb, and TPOAb were associated with anxiety in male patients.

## Introduction

Major depressive disorder (MDD) is characterized by persistent low mood, decreased interest in most activities, weight loss, difficulty sleeping and concentrating [1]. The World Health Organization reports that approximately 350 million people worldwide suffer from major depression, and the recurrence and mortality rates are quite high, making it a leading cause of death and disability and a major contributor to the overall global burden of disease, with a heavy socioeconomic burden [2]. MDD is more common in women, with the prevalence of MDD in women about twice that of men [3, 4]. Although the overall gender differences in the prevalence of MDD have been well studied, little attention has been paid to gender differences in the comorbidity of depression.

Anxiety is a common psychiatric disorder that can seriously impair physical and mental health, quality of life, and is strongly associated with increased risk of chronic disease [5]. It has been reported that up to 70% of patients with MDD may also suffer from anxiety [6], which is considered a pressing issue in MDD. Higher relapse rates [7], higher risk of suicide [8], poorer prognosis [9] and higher socioeconomic costs [10] are often associated with MDD with comorbid anxiety.

Metabolic syndrome (Mets) is defined as an aggregated group of manifestations of central obesity, hypertension, impaired glucose metabolism, and dyslipidemia [11]. In 2012, a meta-analysis evaluating the association between MDD and Mets found a bidirectional association between them [12].Vancampfort et al. reported that the prevalence of metabolic syndrome was 58% higher in patients with psychiatric disorders than in the healthy population [13]. In recent years, psychological factors such as anxiety have also been reported to be strongly associated with metabolic syndrome, independent of traditional risk factors [14, 15]. A meta-analysis found that anxious patients had a higher risk of developing metabolic syndrome, diabetes, hypertension, hyperlipidemia, and obesity. Zhong et al. reported a significant positive association between metabolic syndrome and anxiety [14].

Numerous lines of evidence suggest that depression, metabolic syndrome, and anxiety often coexist and may lead to adverse clinical outcomes. Interestingly, this association may be influenced by gender. Gender differences have been found to play an important role in the comorbidity of MDD [16], affecting the course of illness [17], the prevalence of comorbidities, and treatment outcomes [18]. Cao et al. reported that comorbid anxiety was found in up to 63.4% of female patients with MDD, whereas only 36.6% of male patients with MDD developed anxiety [19]. Female patients with MDD were more likely to have anxiety symptoms such as somatoform disorders and suicide attempts, while male patients were more likely to have substance abuse [20]. Second, gender differences in the prevalence and risk factors for metabolic syndrome were also observed, with Toker et al. reporting a 1.94-fold increased risk of metabolic syndrome in women with MDD [21]. Elevated body mass index, lower HDL cholesterol, increased waist circumference, and elevated blood glucose were risk factors for metabolic syndrome in women, whereas elevated blood pressure and triglycerides were risk factors in men [22]. As gender differences are prominent in both mood disorders and metabolic syndrome, a detailed study of them may help us to understand the mechanisms underlying the onset and maintenance of affective and metabolic disorders. However, gender differences and factors influencing the development of anxiety symptoms in patients with MDD and comorbid Mets are unknown. Therefore, there is an urgent need to develop gender-specific interventions to improve mental health in Chinese populations with comorbid metabolic disorders.

First-episode and drug-naïve (FEDN) patients can minimize confounding factors and examine anxiety status in MDD patients with comorbid Mets without antidepressant exposure, providing the opportunity to conduct our study. In this study, we investigated gender differences in anxiety comorbidity in FEDN MDD patients with comorbid Mets in a Chinese Han population (*N* = 794). The aims of this study were to address (1) whether there are gender differences in demographic characteristics and clinical presentation; and (2) risk factors for anxiety comorbidity in patients with FEDN MDD with Mets.

## Method

### Subjects

A cross-sectional design was used in this study. We recruited 794 patients with MDD and comorbid Mets, including 239 male patients and 555 female patients, from a psychiatric clinic of a general hospital in Shanxi Province, China. The study was approved by the Institutional Review Board (IRB) of the First Clinical School of Shanxi Medical University, and all participants signed an informed consent form.

Participants included in this study had to satisfy the following criteria: (1) aged between 16 and 65 years, Han Chinese population; (2) diagnosed with acute onset MDD according to the Diagnostic and Statistical Manual, Fourth Edition (DSM-IV). Current first episode of depressive symptoms and not receiving any antidepressant, antipsychotic, or other psychotropic medication.

Exclusion criteria included: (1) diagnosis of a psychiatric disorder other than MDD, comorbid serious physical illness, and personality disorder; (2) current pregnancy or lactation; (3) history of substance abuse or dependence (other than nicotine) within the past 6 months; (4) refusal to participate in the study; (5) inability to be interviewed due to an acute clinical condition; (6) using of non-psychotropic drugs (e.g., corticosteroids) that may affect mood and metabolic syndrome; and (7) other unknown reasons.

The definition and diagnosis of Mets is based on the International Diabetes Federation (IDF) consensus criteria for different ethnic groups and includes the following aspects: central obesity, blood pressure, triglycerides, high-density lipoprotein cholesterol, and fasting glucose. Patients diagnosed with Mets had large waist circumference (≥ 90 cm in Chinese men and ≥ 80 cm in women) and two or more of the following: (1) triglycerides ≥ 150 mg/dL; (2) HDLC < 40 mg/dL in men and < 50 mg/dL in women; (3) systolic blood pressure ≥ 130 mm Hg and/or diastolic blood pressure ≥ 85 mm Hg, or have received medication; (4) fasting blood glucose ≥ 100 mg/dL, or have been diagnosed with type 2 diabetes. Other metabolic abnormalities included overweight (body mass index ≥ 25 kg/m^2^), hypercholesterolemia > 200 mg/dL, HDL cholesterolemia > 120 mg/dL, elevated HbA1c (> 5.9%), fasting insulin (> 24.9 uU/mL), and insulin resistance (HOMA-IR > 3) [23].

### Demographic and clinical assessment

A questionnaire was administered to each participant to collect demographic information, including gender, age, education, marital status, age of onset, and duration of illness. Specially trained researchers reviewed the participants’ current medical records.

The Hamilton Rating Scale for Depression (HAMD) was used to assess whether subjects had depressive symptoms [24]. The HAMD scale consists of 17 items, 9 of which are rated on a 3-point scale (0: nonexistent, 2: severe), and 8 of which are rated on a 5-point scale (0: nonexistent, 4: severe). In this study, a HAMD score equal to 24 was the threshold for determining whether participants had or did not have depressive symptoms (Severity classification on the Hamilton depression rating scale).

The presence or absence of anxiety symptoms was assessed by the Hamilton Anxiety Rating Scale (HAMA) [25]. This is the most commonly used measure of anxiety in depression research [26].Patients with a HAMA score of more than 18 were considered to have anxiety symptoms [27].

The above information was collected by two experienced psychiatrists with specialized training. After repeated assessments, the observer correlation coefficients for both HAMD scores and HAMA scores were greater than 0.8.

### Blood samples

Physical parameters including height, weight, waist circumference and blood pressure were checked by research nurses. Biochemical markers were tested on admission before the patients received any treatment. Patients began fasting the night before and serum and plasma samples were collected between 7 and 9 a.m. the next morning.

Fasting blood biochemicals were measured by the hospital testing center and included total cholesterol (TC), triglycerides (TG), high-density lipoprotein cholesterol (HDLC), low-density lipoprotein cholesterol (LDLC), fasting blood glucose (FBG), hemoglobin A1c (HbA1c), and insulin. Insulin resistance was expressed by the Homeostasis Model Assessment of Insulin Resistance (HOMA-IR), which was calculated as: fasting insulin × fasting blood glucose (nmol/L)/22.5.

TSH, FT3, FT4, TPOAb, and TgAb were detected by chemiluminescent immunoassay using a Cobas E610 (Roche, Basel, Switzerland). The lower limits of detection were 0.01 mIU/L, 0.2 pmol/L, and 0.3 pmol/L for TSH, fT3, and fT4, respectively. the inter- and intra-assay coefficients of variation were 5–9% or 3–6%, respectively. The inter- or intra-assay coefficients of variation ranged from 5 to 9% or 3–6%, respectively.

All samples and clinical data were unknown to the technicians who performed the experiments.

### Statistical analysis

For descriptive analysis (conformity to normal distribution), qualitative variables were presented as numbers (percentages) and quantitative variables as means ± SD (Kolmogorov-Smirnov one-sample test, all P’s > 0.05). Second, to observe differences in demographic and clinical variables between men and women, ANOVA and chi-square tests were used to calculate continuous-type variables (including age, age of onset, disease duration, HAMD, and HAMA) and subtype variables (including education and marital status), respectively. Third, to compare gender differences in anxiety symptoms with demographic and clinical parameters, we used a 2 × 2 ANOVA including both gender (2 levels: male and female) and anxiety diagnosis (2 levels: anxiety symptoms and no anxiety symptoms). We tested for effects of gender and anxiety subgroups, and for interactions between gender × anxiety subgroups. We also calculated pearson correlation coefficients to assess the correlation between HAMA scores and other clinical variables. Multiple logistic regression analyses were then performed with HAMA as the dependent variable to assess risk factors for the development of anxiety in patients with MDD and comorbid Mets. All P values were 2-tailed, and the significance level was ≤ 0.05. Bonferroni corrections were used to adjust for multiple comparisons. Statistical analyses were performed using SPSS 21 (SPSS, Inc., Chicago, IL).

## Result

As shown in Table 1, a total of 794 patients with MDD (239 male and 555 female patients) with comorbid metabolic syndrome were recruited in this study. We found that female patients were older and had a higher age of onset compared to male patients. After Bonferroni correction, both age and age of onset remained significant (all *p* = 0.01). However, there were no significant differences between male and female patients in terms of disease duration, HAMD score, HAMA score, education level, marital status, and psychological symptoms (all *p* > 0.05).

The demographic characteristics and clinical data of male and female patients with MDD in anxiety symptom group and no anxiety symptom group are shown in Table 1. Chi-square tests revealed no gender differences in the prevalence of comorbid anxiety in patients with MDD and comorbid Mets (X^2^ = 0.52, *p* = 0.37). ANOVA was then used to examine the interaction between anxiety symptoms and gender. Two-way ANOVA with between-group factors for anxiety diagnosis (level 2, with and without severe anxiety symptoms) and gender (level 2, male and female) revealed that anxiety had a significant effect on duration of illness (F = 6. 48, *p* = 0.01), HAMD score (F = 161.06, *p* < 0.01), HAMA score (F = 715.49, *p* < 0.01), TSH (F = 75.60, *p* < 0.01), TgAb (F = 6.93, *p* = 0.01), and TPOAb (F = 15.64, *p* < 0.01). The analysis also showed that there was a significant gender difference for disease duration (F = 3.95, *p* = 0.04), but no significant effect of gender on other variables. In addition, there was no significant anxiety × gender effect (all *p* > 0.05).

**Table 1:** Clinical features of female and male patients with major depressive disorder with Mets

Variables	Total MetS	Male	Female	$$ {X}^{2}$$	*P* value
Sample Size	794	239	555		
Age (years)	37.50 ± 12.65	35.86 ± 12.55	38.21 ± 12.63	**2.45**	**0.01**
Duration of disease (months)	6.91 ± 4.91	7.19 ± 5.54	6.79 ± 4.61	0.09	0.93
Age of onset (years)	37.25 ± 12.52	35.59 ± 12.36	37.99 ± 12.52	**2.53**	**0.01**
HAMD	31.22 ± 2.86	31.33 ± 2.88	31.17 ± 2.85	0.45	0.65
HAMA	21.51 ± 9.79	21.41 ± 3.52	21.55 ± 3.75	0.44	0.66
Education				4.092	0.25
Junior high school, *n* (%)	244 (30.73)	66 (27.62)	178 (32.07)		
High school, *n* (%)	333 (41.94)	98 (41.00)	235 (42.34)		
University degree, *n* (%)	175 (22.04)	63 (26.36)	112 (20.18)		
Master’ s degree, *n* (%)	42 (5.29)	12 (5.02)	30 (5.41)		
Marital status				1.64	0.20
Single, *n* (%)	186 (23.43)	63 (26.36)	123 (22.16)		
Married, *n* (%)	608 (76.57)	176 (73.64)	432 (77.84)		

Note:

HAMD = Hamilton depression rating scale;

HAMA = Hamilton anxiety rating scale;

Next, we also compared data from male and female patients separately to examine the relationship between HAMA scores and demographic data and clinical indicators. The results are shown in Table 2; in female patients with MDD and comorbid Mets, HAMA score was strongly associated with HAMD score, TSH, TgAb, and TPOAb (*p* < 0.01). In male patients, however, HAMA scores were only strongly correlated with TSH, TgAb, and TPOAb (*p* < 0.01).

**Table 2:** Demographic and clinical characteristics between MDD patients with and without anxiety symptoms, grouped by gender

Variables	With anxiety (*N* = 138)		Without anxiety (*N* = 656)		Gender F (p)	Subgroup F (p)	Gender* subgroup F (p)
	Male	Female	Male	Female			
Sample size	38	100	201	455			
Age (years)	38.53 ± 11.35	38.58 ± 12.85	35.35 ± 12.72	38.13 ± 12.59	1.15 (0.28)	1.89 (0.16)	1.06 (0.30)
Duration of disease (months)	9.03 ± 5.81	7.13 ± 4.43	6.84 ± 5.43	6.71 ± 4.64	**3.95 (0.04)**	**6.48 (0.01)**	3.02 (0.08)
Age of onset (years)	38.15 ± 11.16	38.34 ± 12.71	35.10 ± 12.54	37.90 ± 12.49	1.31 (0.25)	1.79 (0.18)	1.01 (0.31)
HAMD	34.53 ± 2.41	33.65 ± 2.43	30.73 ± 2.54	30.62 ± 2.63	3.31 (0.06)	**161.06 (0.00)**	2.07 (0.15)
HAMA	27.21 ± 1.64	27.42 ± 2.07	20.31 ± 2.58	20.26 ± 2.63	0.090(0.76)	**715.49 (0.00)**	0.24 (0.61)
TSH, µIU/L	7.74 ± 2.11	8.47 ± 2.36	5.98 ± 2.35	5.95 ± 2.37	2.05 (0.15)	**75.60 (0.00)**	2.37 (0.12)
TgAb, IU/L	163.55 ± 371.05	160.78 ± 324.47	87.48 ± 200.23	100.45 ± 235.48	0.03 (0.84)	**6.93 (0.01)**	0.09 (0.76)
TPOAb, IU/L	203.17 ± 350.12	134.79 ± 262.49	77.76 ± 150.90	90.35 ± 196.84	1.68 (0.19)	**15.64 (0.00)**	3.55 (0.06)
FT3, pmol/L	4.90 ± 0.84	4.91 ± 0.70	4.97 ± 0.69	4.89 ± 0.70	0.20 (0.60)	0.11 (0.73)	0.38 (0.53)
FT4, pmol/L	17.45 ± 2.86	16.78 ± 3.02	16.60 ± 3.14	16.50 ± 3.09	1.42 (0.23)	3.09 (0.07)	0.76 (0.38)

Note:

HAMD = Hamilton depression rating scale; HAMA = Hamilton anxiety rating scale; TSH: Thyroid Stimulating Hormone; TgAb: Thyroglobulin antibody; TPO-Ab: Thyroid peroxidase

Bold indicate significant correlations between components

Risk factors associated with the development of anxiety symptoms in patients with MDD and comorbid Mets were further explored by multiple logistic regression analysis. The results showed that in male patients, the following variables were independently associated with anxiety symptoms: TSH (B = 0.372, t = 2.307, *p* = 0.035) and TgAb (B = 1.021, t = 2.802, *p* = 0.006). In female patients, the following variables were independently associated with anxiety symptoms: age of onset (B = 1.594, t = 2.026, *p* = 0.046) and HAMD score (B = 0.389, t = 3.949, *p* < 0.001) (Tables 3 and 4).

**Table 3:** The relationship between HAMA and other clinical data in male and female patients

Variables	Male (*N* = 38)		Female (*N* = 100)	
	*r*	*p*	*r*	*p*
Age (years)	0.07	1.00	0.13	1.00
Duration of disease (months)	-0.29	1.00	-0.05	-1.00
Age of onset (years)	0.09	1.00	0.14	1.00
HAMD score	0.32	1.00	**0.43**	**< 0.01**
TSH, µIU/L	0.30	**< 0.01**	0.47	**< 0.01**
TgAb, IU/L	0.18	**< 0.01**	0.19	**< 0.01**
TPOAb, IU/L	0.21	**< 0.01**	0.10	**< 0.01**

Note:

HAMD = Hamilton depression rating scale; TSH: Thyroid Stimulating Hormone; TgAb: Thyroglobulin antibody; TPO-Ab: Thyroid peroxidase

Bold indicate significant correlations between components

**Table 4:** Risk factors associated with the development of anxiety symptoms in male patients with MDD and comorbid Mets

Variables	Coefficients	Std.error	t	*P* value	95% confidence interval for EXP (B)	
	EXP (B)				Lower	Upper
(constant)	22.596	5.171	4.369	0.000	11.923	33.269
Age (years)	0.569	1.289	0.441	0.665	-2.164	3.301
Duration of disease (months)	-0.108	0.145	-0.747	0.466	-0.415	0.198
Age of onset (years)	-0.558	1.280	-0.436	0.669	-3.271	2.156
HAMD	0.135	0.126	1.515	1.068	0.301	-0.133
TSH, µIU/L	**0.372**	**0.161**	**2.307**	**0.035**	**0.030**	**0.714**
TgAb, IU/L	**1.021**	**0.364**	**2.802**	**0.006**	**0.296**	**1.747**
TPOAb, IU/L	0.001	0.001	0.996	0.334	-0.001	0.002

Note:

HAMD = Hamilton depression rating scale; TSH: Thyroid Stimulating Hormone; TgAb: Thyroglobulin antibody; TPO-Ab: Thyroid peroxidase

Bold indicate significant correlations between components

**Table 5:** Risk factors associated with the development of anxiety symptoms in female patients with MDD and comorbid Mets

Variables	Coefficients	Std.error	t	*P* value	95% confidence interval for EXP (B)	
	EXP (B)				Lower	Upper
(constant)	24.278	4.895	4.960	0.000	14.548	34.008
Age (years)	-1.556	0.783	-1.987	0.050	-3.115	0.003
Duration of disease (months)	0.100	0.074	1.347	0.182	-0.048	0.248
Age of onset (years)	**1.594**	**0.787**	**2.026**	**0.046**	**0.028**	**3.160**
HAMD	**0.389**	**0.099**	**3.949**	**0.000**	**0.193**	**0.586**
TSH, µIU/L	0.166	0.114	1.447	0.152	-0.062	0.393
TgAb, IU/L	0.001	0.001	1.656	0.101	0.000	0.002
TPOAb, IU/L	-0.001	0.001	-1.093	0.278	-0.002	0.001

Note:

HAMD = Hamilton depression rating scale; TSH: Thyroid Stimulating Hormone; TgAb: Thyroglobulin antibody; TPO-Ab: Thyroid peroxidase

Bold indicate significant correlations between components

## Discussion

To our knowledge, this is the first study to analyze gender differences in the prevalence of anxiety in patients with MDD and comorbid Mets. Our findings include (1) no gender differences in the prevalence of anxiety in patients with MDD and comorbid Mets, but a higher prevalence of anxiety in female patients (100/138, 72.46%), and (2) gender differences in clinical correlates and risk factors for the development of anxiety in patients with MDD and comorbid Mets. In female patients, HAMA scores were strongly correlated with HAMD scores, number of TSH, TgAb, and TPOAb. In male patients, however, HAMA scores were strongly correlated only with TSH and TgAb. Multiple logistic regression analysis showed that HAMD score and age of onset may be risk factors for the development of anxiety in female patients with MDD and comorbid Mets, whereas TSH and TgAb were risk factors for the development of anxiety in male patients.

Contrary to our expectations, our results showed no gender differences in the prevalence of anxiety in patients with MDD and comorbid Mets; Serrano et al. and Graaf et al. both reported a significantly higher prevalence of comorbid anxiety in female patients with MDD than in male patients [28–29]. Epidemiologic surveys in different regions of China, including Beijing [30] and Shanghai [31], have also shown statistically significant differences in the prevalence of these comorbidities between males and females. However, some studies have also shown no significant gender differences in comorbid anxiety in patients with MDD, which is similar to our findings [32–34]. The differences between these studies may be due to the fact that the participants in our study were first-episode and first-treatment patients with MDD, most of whom were not under the influence of various antipsychotic and antidepressant medications. In this study, we also found a significantly higher prevalence of anxiety in female patients with MDD and comorbid Mets than in previous studies. In China, the prevalence of anxiety in women is typically 60–70% [35–36]. Most studies from European countries have reported anxiety prevalence rates of no more than 60% in women, and the prevalence of depression combined with anxiety ranges from 40 to 60% [9]. However, our study showed that the prevalence of anxiety in women with MDD and comorbid Mets was 72.46%, and that women with MDD developed anxiety symptoms only about 7 months after the onset of the disorder, compared with 9 months in men. Lamers et al. found that patients withMDD who have comorbid anxiety symptoms tend to have a longer duration of illness [37].

The reasons for these differences may be attributed to the following: first, in the present study, we focused on patients with MDD and comorbid Mets. Women are more likely to develop metabolic syndrome than men, and the intensity of metabolic disorders in women increases progressively with age [38]. Many studies have reported that the combination of multiple metabolic indicators of dysregulation leads to the persistence of depressive symptoms [39–40], and the more severe the depressive symptoms, the higher the prevalence of anxiety [41–42]. The female patients included in this study were slightly older than the male patients, and therefore the severity of the metabolic syndrome was perhaps higher in the female patients.

Second, the higher incidence of anxiety in women may be a result of the stigma associated with mental illness [43]. Women suffering from metabolic syndrome are often characterized by central obesity, which can trigger a variety of negative psychiatric outcomes, especially low self-image or somatic problems [44]; because female patients are more emotionally sensitive, the negative outcomes resulting from obesity may cause shame, cardiac stress, and low self-esteem in women, resulting in a very high incidence of anxiety disorders.

Third, the mean age of the women with MDD and comorbid Mets included in this study was 38 years, and under the influence of traditional Chinese culture, women in this age group tend to focus more on their families, which means they may be exposed to a number of stressors related to family and marital needs, such as caring for parents and children, marital breakdown, and death of a loved one, which may increase the risk for the emergence of anxiety symptoms [45]. In addition, changes in midlife health, such as the transition to menopause or intermenstrual periods, can also increase a woman’s risk for low mood and anxiety [45]. All of these factors may make women with MDD and comorbid Mets very vulnerable to developing anxiety symptoms soon after the onset of the disease.

Another finding of our current study was that there were gender differences in clinical variables associated with comorbid anxiety. In women with MDD and comorbid Mets, HAMA scores was significantly associated with HAMD scores, which were independent risk factors for anxiety comorbidity [46]. This is consistent with most previous studies, in which Hong et al. reported that women with anxiety had higher HAMD scores [47–48], and both Fava et al. [35] and Lamers et al. [37] found that patients with MDD with comorbid anxiety typically had more severe depressive symptoms and more suicidal ideation. Gender differences in clinical variables associated with comorbid anxiety may be explained by biological factors. Anti-regulatory hormones such as catecholamine neurotransmitters and glucagon, which are activated during psychological stress and metabolic syndrome, can lead to disturbances in glucolipid metabolism and abdominal obesity [49–50]. The side effects of this poorly controlled metabolic disorder may lead to the persistence of depressive symptoms [51]. On the other hand, excessive release of glucocorticoids during stress leads to a relative lack of neutralization by normal levels of estrogen, leaving women more vulnerable to stress and therefore at higher risk for anxiety and depression [52–53]. Based on this finding, we hypothesize that women with metabolic disorders may be more susceptible to emotional control, particularly anxiety, than men.

We also found that the clinical variables independently associated with anxiety comorbidity in male patients with MDD and comorbid Mets were TSH and TgAb levels, indicating that both were independent risk factors for comorbid anxiety in male patients, which is consistent with previous studies. Yang et al. reported that patients with MDD with abnormally elevated TSH were more likely to have comorbid anxiety [54]. Both anxiety disorders, including panic disorder and social anxiety disorder, and depression are associated with abnormal thyroid function, such as elevated serum TSH and TgAb levels [55–56]. However, some studies have reported inconsistent results. For example, Ittermann et al. reported that TSH levels were not significantly associated with anxiety [55]. A recent study found an inverse relationship between TSH and self-reported anxiety [57]. According to our results, elevated serum TSH and TgAb levels can be used as potential indicators of comorbid anxiety in male patients with MDD and comorbid Mets, but need to be validated in future studies.

This study has several limitations: first, this cross-sectional study could not explain the causal relationship between MDD, Mets, and anxiety, which needs to be confirmed in a future prospective cohort study. Second, the high number of female patients included in this study may be a potential confounder, as women have a higher prevalence of anxiety. In the future, rigorous adjustment for relevant confounders is needed where appropriate. Third, smoking and alcohol consumption were not included in this study, and these variables should be controlled to exclude their influence on the relationship between the three to make the study more accurate. Fourth, our study included only patients with FEDN; however, most patients may be taking a variety of antipsychotic and antidepressant medications; therefore, the results of this study cannot be extrapolated to other settings. Fifth, there are many factors associated with anxiety, such as family caregiving and level of physical health, but these factors were not uncontrolled in the adjusted analysis.

In conclusion, there was no gender difference in the prevalence of anxiety in patients with MDD and comorbid Mets. HAMD score and age of onset were associated with the prevalence of anxiety in female patients, whereas they were associated with TSH, TgAb, and TPOAb in male patients.

## Data Availability

Data will be available from the corresponding author on reasonable request.
